# Asymptomatic costal exostosis with thickening in the pericardium: a case report

**DOI:** 10.1186/s13019-016-0431-1

**Published:** 2016-03-05

**Authors:** Toru Kameda, Takashi Makino, Takamitsu Sakai, Satoshi Koezuka, Hajime Otsuka, Yoshinobu Hata, Naobumi Tochigi, Kazutoshi Shibuya, Akira Iyoda

**Affiliations:** Division of Chest Surgery, Toho University School of Medicine, Tokyo, Japan; Department of Surgical Pathology, Toho University School of Medicine, Tokyo, Japan

**Keywords:** Exostosis, Rib, Asymptomatic, Surgical indication

## Abstract

**Background:**

Costal exostosis is a benign condition that sometimes requires emergent surgery because of associated hemothorax; in addition, there have been cases with malignant transformation to chondrosarcoma. Here, we describe an asymptomatic patient who underwent thoracoscopic resection for primary costal exostosis.

**Case presentation:**

A 16-year-old male was found to have a bow-shaped shadow on a chest X-ray. Chest computed tomography revealed a rod-like mass with a soft tissue shadow adjacent to the left fifth rib. A thoracoscopic partial resection of the left fifth rib was performed. Intraoperative findings included thickening of the pericardium near the tip of the growth and erosion of the visceral pleura of the left lung. The resected specimen was diagnosed as a primary costal exostosis based on histopathological findings.

**Conclusions:**

We review the published literature on costal exostosis and discuss the surgical indications of asymptomatic cases.

## Background

A costal exostosis is a benign growth capped by cartilage, which protrudes from a rib. It may sometimes require emergent surgery because of an associated complication such as damage to an intrathoracic organ [1, 2], and there have been some cases that have transformed to chondrosarcoma [3]. We performed a resection of an asymptomatic primary costal exostosis that had been incidentally discovered on a chest X-ray and I found the exostosis was accompanied with wall thickening in the pericardium and visceral pleura of the left lung. We discuss the indications for surgery for patients with costal exostosis, focusing on asymptomatic cases.

## Case report

**Fig. 1:**
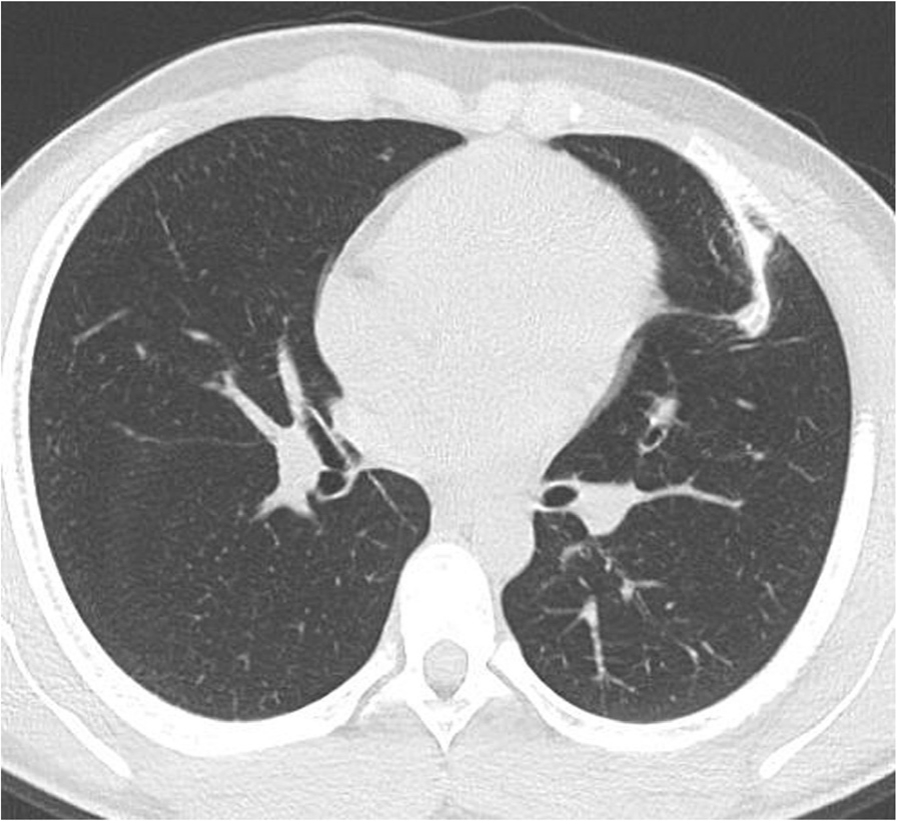
Chest computed tomography scan shows a rod-like mass with a soft tissue shadow adjacent to the left fifth rib

A 16-year-old male was found to have a bow-shaped shadow to the left of the middle lung field on chest X-ray. Chest computed tomography (CT) revealed a rod-like mass with a soft tissue shadow adjacent to the left fifth rib (Fig. 1). The diagnosis was primary costal exostosis of the left fifth rib, and the growth was resected because of the risk of damage to intrathoracic organs.

**Fig. 2:**
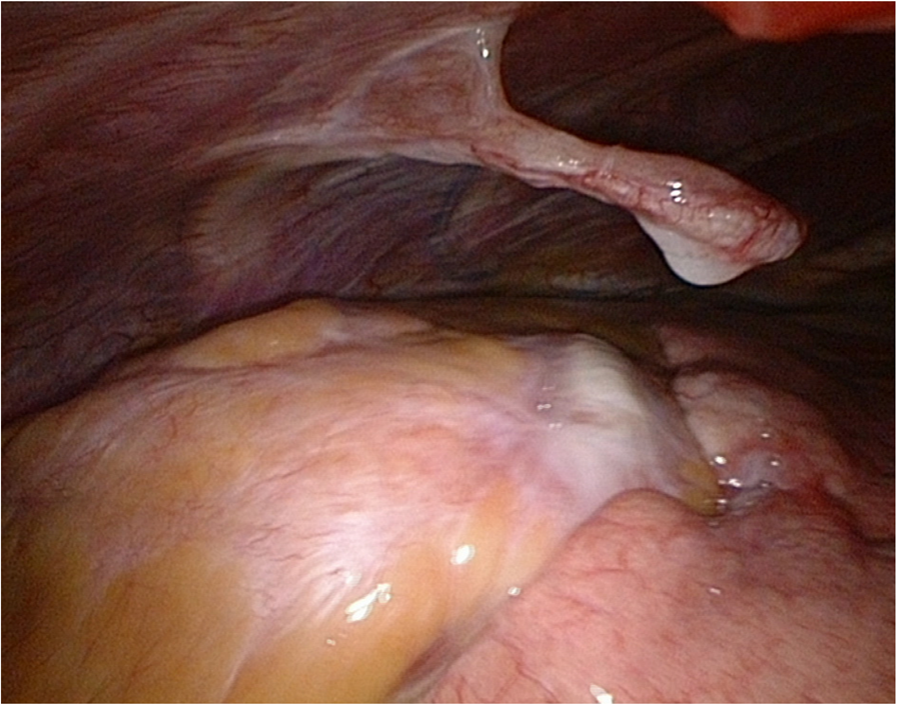
View through the thoracosope shows an approximately 50-mm long structure protruding from the fifth rib into the chest cavity. Thickening of the pericardium near the tip of the structure and erosion of the visceral pleura of the left lung are also visible

The patient underwent thoracoscopic partial resection of the left fifth rib. An approximately 50-mm long structure protruding from the fifth rib into the chest cavity was observed through the thoracoscope. Thickening in the pericardium near the tip of the structure and erosion of the visceral pleura of the left lung were also seen (Fig. 2). The cartilage cap could not be identified, and it was resected with the fifth rib.

Histopathological examination of the resected specimen found that the growth was composed of mature bone tissue; there were no atypical cells or bone marrow within the growth. No cartilage cap was found, but thickened fibrotic tissue was observed and thought to represent inflammatory changes caused by degeneration and omission or mechanical irritation of the cartilage cap. There were no signs of malignant changes, and the growth was diagnosed as a primary costal exostosis.

## Discussion

Exostosis has accounted for 50 % of primary benign bone tumors and has been observed to occur at the metaphysis of long bones or originate from the surface of flat bones [4]. It generally occurs at the proximal femoral or distal tibial metaphysis [1]. Exostosis of the rib makes up a small proportion of the cases of exostosis [4].

Primary exostosis occurs as solitary or multifocal lesions, and multiple exostosis is a hereditary disease that occurs in infants [5, 6]. Hereditary multiple exostosis is a rare disorder characterized by the formation of exostoses in many locations. It is associated with mutations in the *EXT* genes and is manifested by skeletal deformities caused by abnormal bone growth [5].

Solitary exostosis occurs in both infants and adults [1, 4], and is generally asymptomatic. When an X-ray diagnosis of an asymptomatic case is not definitive, a chest CT is useful. Symptomatic cases of costal exostosis have presented with swelling, hiccup [7], chest pain [7], pneumothorax [4, 8, 9], or hemothorax [1, 2]. Hemothorax is thought to result from trauma caused by the costal exostosis to the pleura [6], diaphragm [1, 10], lung [11, 12], or heart [13]. Cases at risk of life-threatening damage to intrathoracic organs should be resected surgically.

Past reports of organ damage due to costal exostosis have described longitudinal lacerations of the diaphragm and pericardium adjacent to the tip of the exostosis that were caused by movements during respiration. Even in cases such as ours, with asymptomatic anterior left fifth costal exostosis, histopathological changes were seen in the pericardium and lung. In a patient with anterior left fourth rib exostosis, thickened pericardium and visceral pleura possibly caused by scratch have been reported [14]. Although in our patient the distance between an intrathoracic organ and the exostosis was long, life-threatening damage to an intrathoracic organ due to respiratory movements was possible. Even if the tip of protrusion seems apart enough from the pericardium or diaphragm, anterior costal exostosis can come in touch with these intrathoracic organs during the movement of the chest wall, resulting in just a scratch or a fatal injury according to the strength of the external pressure. Therefore, preventive surgical treatment should be considered especially for the anterior left costal exostosis.

Malignant transformation of an exostosis to chondrosarcoma [15] is relatively rare; the risk of a solitary exostosis transforming to chondrosarcoma is 1–2 %, and for multiple osteocartilaginous exostoses is 5–25 % [3]. Chondrosarcoma should be suspected if a lesion continues to grow in the patient after puberty or localized pain develops [4].

Because of the risks of hemothorax, other life-threatening complications, and malignant transformation, prophylactic surgical resection should be considered for patients with costal exostosis. Care should be taken to perform resections with adequate margins in order not to leave the residual cartilage cap, especially for cases where the extent of the cartilage cap cannot be determined, and for cases of exostosis occurring at sites other than the ribs. There have been reports of cases of exostosis involving other bones that have developed postoperative recurrence because the initial resection was inadequate.

### Informed consent

Written informed consent was obtained from the patient for publication of this case report and any accompanying images. A copy of the written consent is available for review by the Editor-in-Chief of the *Journal of Cardiothoracic Surgery*.

## Conclusions

We reviewed the published literature on costal exostosis and discussed the surgical indications of asymptomatic cases. Prophylactic surgical resection should be considered for patients with costal exostosis.
